# Implementation of Aging Mechanism Analysis and Prediction for XILINX 7-Series FPGAs with a 28-nm Process

**DOI:** 10.3390/s22124439

**Published:** 2022-06-12

**Authors:** Zeyu Li, Zhao Huang, Quan Wang, Junjie Wang, Nan Luo

**Affiliations:** School of Computer Science and Technology, Xidian University, Xi’an 710071, China; zeyuli@stu.xidian.edu.cn (Z.L.); qwang@xidian.edu.cn (Q.W.); junjiewang@stu.xidian.edu.cn (J.W.); nluo@xidian.edu.cn (N.L.)

**Keywords:** FPGA, aging mechanism, NBTI, measurement error correction, aging prediction, machine learning

## Abstract

Commercial off-the-shelf (COTS) field-programmable gate arrays (FPGAs) with a 28-nm process have become popular devices for computing systems. Although current generation FPGAs have advantages over previous models, the phenomenon of circuit aging has become more significant with the sharp reduction in the process size of FPGAs. Aging results in FPGA performance degradation over time and, ultimately, hard faults. However, few studies have focused on understanding aging mechanisms or estimating the aging trend of 28-nm FPGAs. For this, we used a ring oscillator (RO)-based test structure to extract data and build a dataset that could be used to predict aging trends and determine the primary aging mechanisms of 28-nm FPGAs. Moreover, we proposed a correction method to correct temperature-induced measurement errors in accelerated tests. Furthermore, we employed four machine learning (ML) technologies that were based on accurate measurement datasets to predict FPGA aging trends. In the experiment, 24 XILINX 7-series FPGAs (28 nm) were evaluated for 10+ years of circuit operation using accelerated tests. The results showed that the aging effects of negative-bias temperature instability (NBTI) was the primary aging mechanism. The correction method proposed in this paper could effectively eliminate measurement errors. In addition, the minimum prediction error rate of the ML model was only 0.292%.

## 1. Introduction

Commercial off-the-shelf (COTS) field-programmable gate arrays (FPGAs) with a 28-nm process have become popular devices for computing systems. Although current generation FPGAs have advantages over previous models, the continuous scaling of devices to deep nanotechnology and the inexorable reduction in supply voltage significantly challenge the reliability assurance that is related to device aging [1,2,3]. Aging results in FPGA performance degradation over time and, ultimately, hard faults. Hence, it is essential to understand the main aging mechanisms of FPGAs [4,5,6]. Meanwhile, estimating the aging trends of age-related faults before they occur is crucial for developing aging prevention/mitigation actions to avoid circuit failures [7,8].

To effectively solve the above problems, many efforts have been devoted to aging tests for the analysis of aging mechanisms and the prediction of aging trends of FPGAs. The increase in path delay is the primary indicator of FPGA aging degradation. Hence, measuring the variations in path delay can quantify the aging degree of a circuit. For a long time, actual on-chip measurements and sensor-based aging monitoring have been the mainstream methods [2,9,10,11,12,13,14]. Almost all of these methods employ ring oscillator (RO)-based circuits to measure path delay. However, the test processes of the above methods easily affect the measured delay results and cause errors. Therefore, it is essential to correct measurement errors to obtain accurate data. In terms of aging prediction, most studies have used physical aging models to simulate the aging degradation of transistors or look-up tables (LUTs) [15,16,17]. However, the parameters of such models are difficult to determine. Furthermore, some studies have predicted the aging of circuits based on machine learning (ML) [18,19]. Nevertheless, these methods only focus on predicting the path delay degradation that is related to bias temperature instability (BTI).

To make up for the limitations of previous research, we performed an on-chip, accelerated aging test to obtain the aging-related data of 28-nm FPGAs. Meanwhile, we also improved a measurement method to correct measurement errors that were caused by the accelerated experiment. Based on the above work, on the one hand, we investigated the primary aging mechanisms of 28-nm FPGAs; on the other hand, we employed a variety of ML technologies to predict the aging trends of FPGAs. In summary, we achieved the following novel contributions:

- We performed an on-chip, accelerated aging test to observe the effects of different stress signals and LUT configurations on FPGA aging, which showed how the frequencies of ROs change with aging and which aging mechanisms mainly affect 28-nm FPGAs;
- A measurement method was improved to correct measurement errors that were caused by the accelerated experiment and the corrected data were used for the analysis of the aging effects and the training of the aging prediction model;
- A variety of machine learning technologies were employed to predict the aging trends of FPGAs to evaluate the effectiveness of the ML models for the prediction of FPGA aging trends.
- The experimental results, based on a group of 28-nm XILINX 7-series FPGAs, showed that negative BTI (NBTI) was the main aging mechanism; moreover, the correction method proposed in this paper could effectively rectify measurement errors and in terms of aging prediction, the XGBoost-based ML model was competent for fitting the actual aging trends of FPGAs.

The structure of this paper is organized as follows. In Section 2, we review the important aging mechanisms of ICs and describe related works on aging tests and the aging prediction of FPGAs. Section 3 presents the aging test implementation using FPGAs and proposes the error correction method. The experimental results are presented in Section 4, followed by the conclusion in Section 5.

## 2. Background and Related Work

### 2.1. Aging Mechanisms

Circuit aging refers to the degradation of some of the characteristic hardware parameters in integrated circuits (ICs) over time. It can be summarized as the increase in threshold voltage that is caused by transistor aging, which eventually leads to transistor failure, and the increase in resistance that is caused by metal wire aging, which eventually leads to fracture. The aging mechanisms of transistors and interconnects are dominated by four main effects at the nanoscale: bias temperature instability (BTI), hot carrier injection (HCI), time-dependent dielectric breakdown (TDDB), and electromigration (EM).

BTI is considered to be the main limiting factor of the lifetime of nanoscale complementary metal oxide semiconductor (CMOS) devices and is divided into positive and negative BTI (PBTI/NBTI) [20,21]. HCI is due to the strong channel electric fields near the drain in the channel, which causes the carriers to cross the Si–SiO${}_{2}$ barrier and inject into the oxide medium to form traps and results in the degradation of the threshold voltage [22,23]. TDDB causes local tunnel breakdown and eventually causes dielectric breakdown, which usually leads to catastrophic hard failure. EM is a mechanism that affects the interconnects and induces open circuits (due to voids) or short circuits (due to hillocks).

### 2.2. Aging Tests on FPGAs

FPGA aging degradation can manifest itself as an increase in the probability of transient/permanent failures [24] or as a change in timing. One of the most intuitive and easily observable indications is the increase in the path delay of the circuit. Hence, measuring the variations in path delay can quantify the aging degree of a circuit. In the early stages, a transition probability-based delay measurement is the primary method that is used [25]. However, the delay data obtained by this method are not accurate enough since they usually evaluate the worst-case path delay.

With the popularization of built-in self-tests (BISTs) in IC tests, actual on-chip measurements and sensor-based aging monitoring have become the mainstream methods [2,9,10,11,12,13,14,26,27]. Naouss et al. [2] established a low-cost test platform to evaluate FPGA reliability, which supports aging delay measurements for multiple FPGAs at the same time. Miyake et al. and Xiang et al. [9,10] proposed a measurement method based on ROs concerning on-chip delay, which is suitable for field testing. Refs. [11,12,13,14] employed aging sensors to monitor the delays in critical circuit paths to evaluate FPGA aging. Almost all of these methods can obtain relatively accurate delay data and their measurements are based on RO circuits. Hence, this study also employed RO-based measurement circuits to test FPGA aging.

### 2.3. Aging Prediction of FPGAs

Most early studies used physical aging models to predict the aging degradation of transistors and LUTs. Morales et al. [15] developed a general simulation environment to implement FPGA circuits that can predict the LUT propagation delay of digital circuits. Jang et al. [16] proposed an on-chip aging sensor circuit to predict and detect circuit failures caused by the effects of BTI and HCI aging on digital circuits. Yu et al. [17] proposed a fast time-zero aging prediction and predictive screening methodology based on a novel on-chip architecture, named ZeroScreen. However, the implementation of the above methods usually depends on the transistor or LUT model. Therefore, it is difficult to determine the appropriate formula parameters.

To date, some studies have predicted the aging of circuits based on ML [18,19]. For example, Karimi et al. [18] proposed a general-purpose IC aging prognosis approach that considers a comprehensive set of IC operating conditions, including workload, usage time, and operating temperature. Vijayan et al. [19] proposed a method to perform low-cost and fine-grained workload-induced stress monitoring for accurate age-induced delay prediction. However, these methods only focus on predicting the degradation of BTI-related path delay. In addition, they also have to depend on logic simulation to obtain characteristic values and labels as inputs for the prediction model. In contrast, our method directly exploits the measured data to train the ML-based aging prediction model. As a result, we could predict FPGA aging without depending on physical aging models.

## 3. Aging Test Implementation for FPGAs

### 3.1. Design of Test Solution

In this study, the on-chip aging test was performed using an RO circuit. Due to the self-oscillation characteristics of the RO, the change in its frequency could characterize the aging degradation of FPGAs.

**Figure 1:**
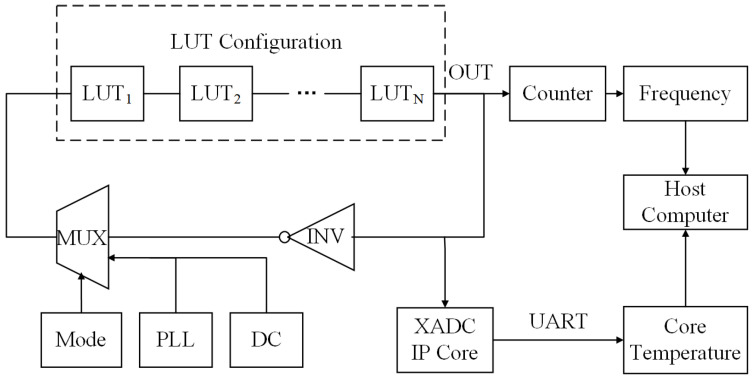
RO-based test structure.

Figure 1 shows the RO-based structure. The test circuit had two working modes: accelerated aging mode (0) and test mode (1). The user sent the status-control bit signal to the circuit through the UART when switching modes. When mode = 1, the circuit was in an open-loop state to accelerate its aging under test conditions by inputting a signal of a specific waveform as a stress signal. This could be a static signal (DC0, DC1) or a signal that was generated via a PLL of the FPGA, for which the user determined the frequency and duty cycle. When mode = 0, the aging state was be measured. As the circuit was in a closed-loop condition at this point, a measurement method based on the ring oscillator was employed and the counter produced the corresponding frequency. During the test, we also used the XADC IP core [28] to periodically monitor the core temperature and analyze the influence of temperature on measurement errors.

**Figure 2:**
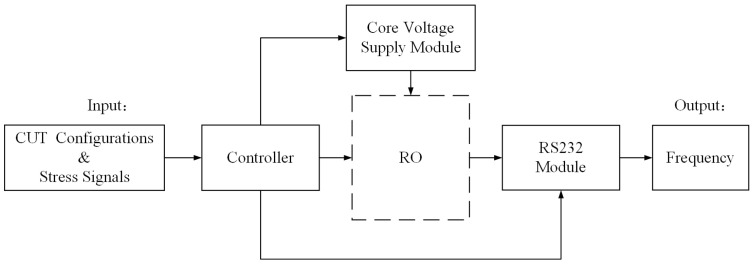
Logic function module of the aging test.

The logic function of the test is shown in Figure 2. The core of the test was the controller module, which was responsible for coordinating the whole test process. The core voltage supply module provided the required working voltage for the FPGA. The RS232 module was responsible for the communication between the FPGA and the PC. The input was the configuration file and stress signal of the circuit under test (CUT) and the output was the frequency value of the CUT.

### 3.2. Accelerated Aging Conditions

An accelerated test refers to the accelerated degradation of a tested product by strengthening the test conditions, under the premise of ensuring that the failure mechanisms of the product are not changed, to obtain the necessary information in a relatively short period of time [29]. The aging speed of FPGAs is normally limited and long-term aging tests cannot be achieved within set time parameters. Hence, it is incredibly important to carry out accelerated tests [30]. In line with the principle regarding the aging mechanisms of BTI, HCI, and the theoretical acceleration model, the aging speed was directly related to the working voltage and temperature of the circuit and their relationships could be expressed as in the following formulae [31]:

(1) $$t_{f}\propto V_{gs}^{-\gamma }$$

(2) $$t_{f}\propto exp(\frac{E_{a}}{kT})$$

where $t_{f}$ represents the estimated duration for which the circuit can operate reliably. Based on the evidence from the available work in the literature [31], the value of γ is usually 6–8, $E_{a}$ is approximately 1.5 eV, and *k* is Boltzmann’s constant. *T* stands for the operating temperature of the circuit, while $V_{gs}$ is the gate-source voltage of the transistor.

The objects of this accelerated aging test were XILINX 7-series FPGAs. According to the FPGA manual, the range of supply voltage, without causing damage, is 0.5 V to 1.1 V, the general working voltage is 1.0 V, the working temperature is between $-40{\;}^{\circ }$C and +100 ${}^{\circ }$C, and the general working environment temperature is 27 ${}^{\circ }$C. Based on aging theory and the test conditions, Table 1 presents the theoretical power supply voltages and operating temperatures for the aging tests and the estimates of the acceleration under these conditions.

### 3.3. Correction Method for Measurement Errors

The aging degree of a device is aggravated by increases in operation time, which indicates the cumulative growth of the circuit path delay. The acceleration experiment was carried out in a high-temperature environment and the delay that was measured on-chip was affected by the temperature. Assuming that the initial delay of the circuit was $D_{0}$, the measured value was the sum of the initial delay, the aging delay $D_{aging}$ of the circuit, and the temperature-related error value $D_{error}$:

(3) $$D_{measure}=D_{0}+D_{aging}+D_{error}$$

At this time, the measurement value could not reflect the real delay of the circuit. Thus, the influence of delay variation due to temperature change had to be eliminated to obtain temperature-independent delay measurements. In this regard, we researched the error correction method. It was assumed that the time delay caused by aging would not increase over a concise period, i.e., $\lim_{\Delta t\to 0}\Delta D_{aging}=0$, where $\Delta D_{aging}$ is the delay variation caused by aging. Therefore, the delay variation from sampling at different temperatures was the delay error caused by temperature, i.e., $\Delta D=D_{error}=D_{measure}-D_{0}$. At this time, the correlation coefficient λ=ΔD/ΔT was introduced, which represented the relationship between the change in measurement delay and the change in temperature. When λ was a constant value (i.e., the variation in delay error caused by temperature was in a fixed proportion to the variation in temperature), the measurement error could be corrected by the λ value, correction value $D_{correct}=\lambda \Delta T$, and real aging delay $D_{aging}=D_{measure}\pm D_{correct}$. Then, the research focused on computing the value of λ.

**Figure 3:**
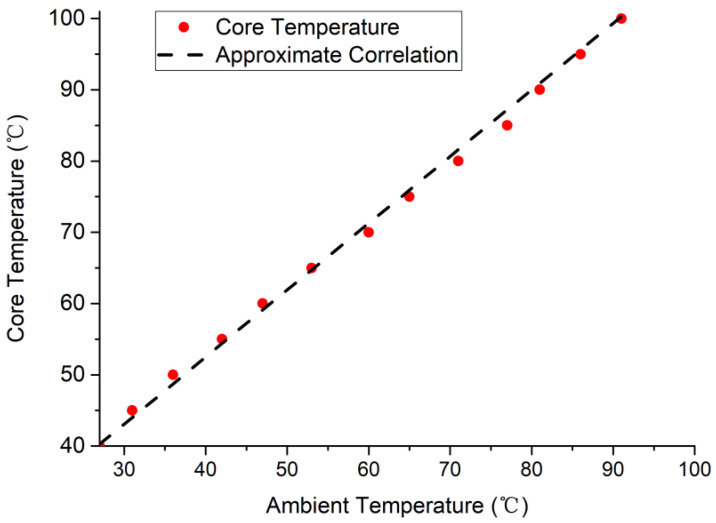
The relationship between the ambient temperature and the core temperature.

To obtain λ, we first measured the initial circuit delay $d_{0}$ and then constantly changed the core temperature (CT) and synchronously measured the change in the on-chip circuit delay. Meanwhile, we recorded the D value between the current and initial temperatures. To reduce the difference that was caused by this process, the experiment was carried out on six FPGAs, with each FPGA running the same CUT. We simultaneously set the temperature, recorded the relevant data, and obtained the average value of λ across the six groups of data. Before that, we also determined the relationship between the external environment temperature (AT) and the CT in order to accurately adjust the core temperature. Figure 3 shows the relationship between the ambient temperature and the core temperature throughout the experimental measurements.

The red circle in Figure 3 represents the measured CT and it can be seen that the CT had a linear relationship with ΔT. It was found that they accorded with CT=0.9375×AT+15 (dotted line) by calculation. It should be noted that the environment was a sealed aging test chamber. When the AT reached 91 ${}^{\circ }$C, the CT could reach 100 ${}^{\circ }$C, which was the upper limit of the CT of the FPGAs. We could adjust the CT accurately, according to the formula. In the experiment, the initial temperature was 27 ${}^{\circ }$C. We adjusted the core temperature from 40 ${}^{\circ }$C to 100 ${}^{\circ }$C in 5-${}^{\circ }$C intervals and calculated the corresponding λ temperature, which was denoted as $\lambda_{1}$~$\lambda_{12}$. The results are shown in Table 2.

It can be seen that the λ values that corresponded to different temperatures obviously changed, which indicated that the correction method mentioned above could not be used directly. We proposed the hypothesis that when the λ value does not change with the increase in duration at the same temperature, the delay variation measured over two time periods is equal to the delay variation that is caused by aging. Then, the actual aging delay could be corrected by calculating the correction value $D_{correct}=\lambda_{T}\times \Delta T$, where $\lambda_{T}$ is the coefficient at the current temperature. To verify the feasibility of this method, we calculated the λ coefficient at different aging times. We designed a 1000-h experiment and calculated the λ coefficients every 100 h. The λ coefficients corresponded to 60 ${}^{\circ }$C ($\lambda_{1}$), 80 ${}^{\circ }$C ($\lambda_{2}$), and 100 ${}^{\circ }$C ($\lambda_{3}$). Similarly, to reduce the difference caused by the process, the experiment was carried out on 10 FPGAs, with each FPGA running the same CUT. We simultaneously set the temperature, recorded the relevant data, and obtained the average value across the ten data groups. The experimental results are shown in Table 3.

It can be seen from the table that there were slight differences (measurement errors) in the measured λ values at different temperatures. Generally speaking, it could be proved that the temperature-dependent delay did not change with the duration increase. In practice, we calculated the corresponding λ value at the temperature that corresponded to the core temperature and then, we could correct any errors in the measurement. By restoring the measurement errors, the accurate aging delay could be obtained by way of on-chip measurements. The chip could be continuously accelerated without waiting for the temperature to return to the average temperature to obtain accurate measurements. It is evident that each heating and cooling process was time-consuming and that the critical data were unstable.

## 4. Test Results and Analysis

### 4.1. Experimental Setup

We used 24 XILINX 7-series FPGAs (28 nm) for the aging tests in our experiment. The host computer was a Xeon(R) Silver 4116 (2.10 GHz) CPU with 32 GB DDR4 RAM, which was running Windows 10. The reconfiguration fabrics of each FPGA were divided into 16 reconfigurable regions to execute the CUTs.

To understand the primary aging mechanisms of 28-nm FPGAs, we combined different stress signals and LUT configurations as the test conditions. In this experiment, five common frequencies (DC0, DC1, 50 MHz, 100 MHz, and 300 MHz) and three duty cycles (DC25, DC50, and DC75) were selected as the combined stress signals and were input into the CUTs. The LUTs of the ROs were configured as BUFFER, AND, XOR, and INV and were executed in each of the four groups of chips. Hence, degradation due to certain test conditions was the mean value of the degradation of the six circuits under test.

**Figure 4:**
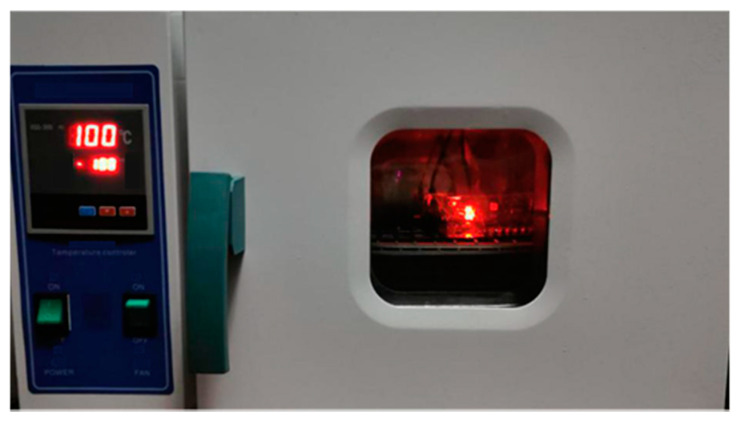
Operation state of the high-temperature test chamber.

The conditions of V and T under this setting were approximately equal in order to eliminate any differences in the manufacturing process. The value of each data point was the average value of the same six CUTs. Moreover, the voltage was provided by external stabilized power and the high temperatures were produced by a 101-0B high-temperature test chamber, as shown in Figure 4, which was capable of providing a stable temperature environment for the test from 50 ${}^{\circ }$C to 300 ${}^{\circ }$C, thus meeting the needs of the accelerated degradation tests.

To evaluate the ML-based models for the application of FPGA aging prediction, we employed four ML technologies (XGBoost, SVM, LR, and ANN) to model the reconfiguration circuits. In the experiments, we extracted the data from all 24 XILINX 7-series FPGAs from the aging tests and aging simulation experiments to build our dataset (frequency, duty cycle, operation time, LUT configuration, delay variation, etc.) and this dataset was then used to train and test the prediction model. We used the root mean squared error (RMSE) as the evaluation metric in this experiment.

### 4.2. Influence of Stress Signals on FPGA Aging

Here, we present the influences of different stress signals on FPGA aging degradation and their analysis to find the primary aging mechanisms of 28-nm FPGAs.

#### 4.2.1. The Influence of Frequency

**Figure 5:**
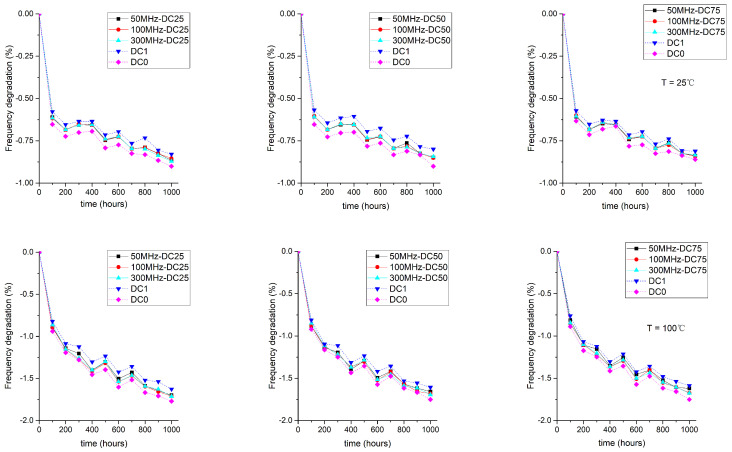
Impacts of stress signal frequency on the frequency degradation of ROs.

Dynamic stresses (50 Mhz, 100 Mhz, and 300 Mhz) and static stresses (DC0 and DC1) that related to different operating frequencies were selected as the inputs for the stress signals. Figure 5 shows the frequency degradation of the ROs under other test conditions. As expected, the degradation that was caused by the NBTI and HCI mechanisms increased as the temperature rose. After 1000 h of accelerated testing, we found that the degradation was 1.8% at a working temperature of 100 ${}^{\circ }$C and 0.9% at a working temperature of 25 ${}^{\circ }$C. However, we did not observe regularity in the aging degradation that was caused by dynamic AC stress. One possible explanation could be that this degradation results from the combined effect of two aging mechanisms: NBTI and HCI. Existing studies have demonstrated that the aging effects of NBTI decrease with increasing stress frequency, while the aging effects of HCI increase with increasing stress frequency [32,33]. When the stress frequency changes, these two aging mechanisms change in opposite directions at the same time. Therefore, it could not be analyzed whether there was a (positive or negative) correlation between the change in frequency and the aging degradation.

#### 4.2.2. The Influence of Duty Cycle

**Figure 6:**
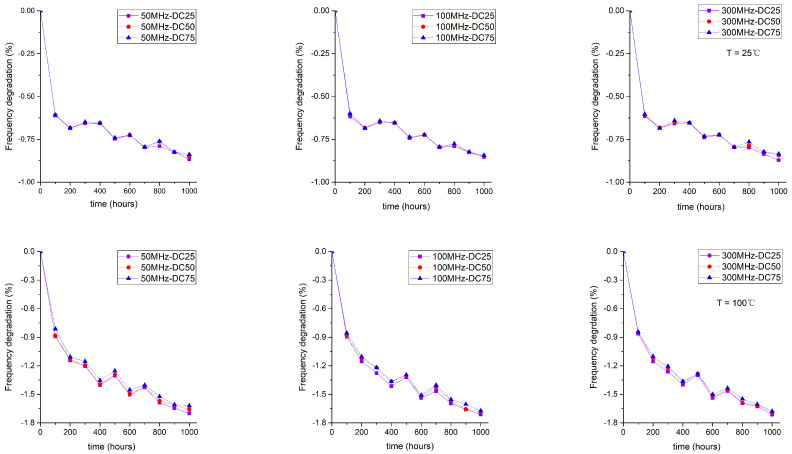
Impacts of duty cycle on the frequency degradation of ROs.

Three duty cycles (25%, 50%, and 75%) were selected as the stress signals input. Figure 6 shows the frequency degradation of the ROs due to different AC stress signals with the different duty cycles. For the same stress signal frequency, the 25% duty cycle had a more significant drop than the 50% duty cycle, while the 50% duty cycle had a more significant drop than the 75% duty cycle. We could see that this difference was more pronounced at higher temperatures (100 ${}^{\circ }$C vs. 25 ${}^{\circ }$C) and at higher stress signal frequencies (300 MHz vs. 10 MHz). This could be explained by the fact that the period of the low-frequency signal was long enough to restore the NBTI aging mechanism to some extent.

### 4.3. Evaluation of Correction Method

**Figure 7:**
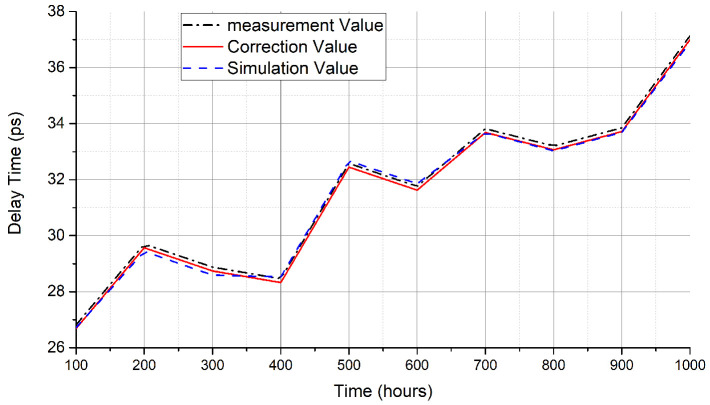
Comparison of the correction delay and simulation delay.

In the experiments, we set the core temperature to 90 ${}^{\circ }$C. We sampled 10 data groups and measured the path delay at 100 h, 200 h, 300 h, 400 h, 500 h, 600 h, 700 h, 800 h, 900 h, and 1000 h. To eliminate measured errors/noise points, the experiment was carried out synchronously on 10 FPGAs and each data point was the mean value across the 10 groups of measured data. After the measurements, we corrected the errors and recorded the corrected data. To evaluate the effectiveness of the correction method, we used ModelSim to simulate the aging of the XILINX 7-series FPGAs and recorded the delay data that corresponded to the simulation time points. As shown in Figure 7, it was found that the difference between the corrected delay and the simulation delay was within 1%, which proved that the correction method was effective.

### 4.4. Results of Aging Prediction

**Figure 8:**
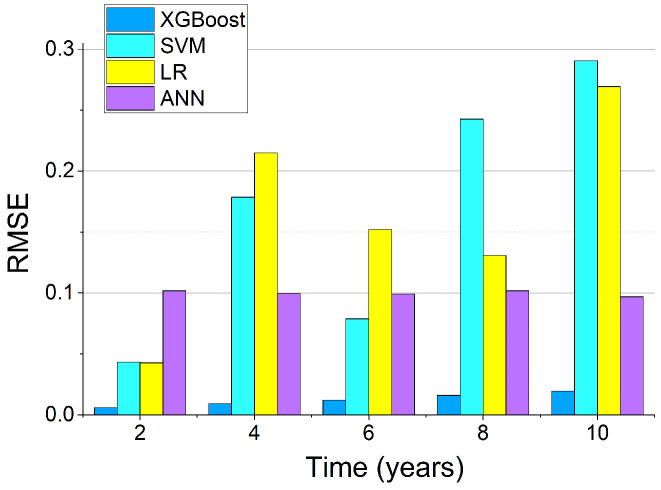
RMSEs of the different ML models.

The results of the RMSE of the XGBoost, SVM, LR, and ANN models are presented in Figure 8. It can be seen that the RMSE of the ANN was very stable, but there were still significant errors when the predicted values of frequency degradation were low. The RMSE of LR and SVM were relatively high and there were also significant prediction errors. Compared to the other three models, the RMSE of XGBoost was minimal. The increase in RMSE was due to the predicted frequency degradation value also increasing, but the prediction error did not change significantly. The mean error rate of the XGBoost prediction was only 0.292%.

**Figure 9:**
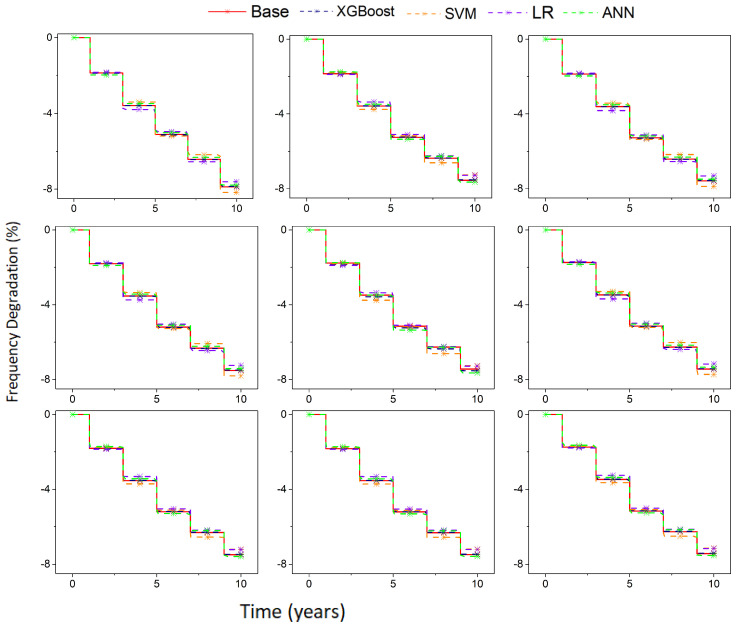
Aging prediction results of the different ML models under different stress signals and LUT configurations.

Figure 9 presents the aging prediction results of the four ML models under different stress signals and LUT configurations of CUTs. The base represents the measured aging degradation. As the results show, the aging trends that were predicted by all ML models were similar to the actual aging trends (red), particularly the prediction of the XGBoost model, which almost entirely coincided with the actual aging trend. Hence, the above experiments illustrated that it would be very feasible to use the ML models to predict the aging degradation of FPGAs.

### 4.5. Discussion

With the shrinking CMOS manufacturing process, NBTI has proven to be the most important aging mechanism. While existing studies have validated this conclusion by performing aging tests on XILINX ARTIX7 FPGAs, we attempted to further validate this conclusion by performing aging tests on a larger number/type of XILINX 7-series FPGAs. The experiments in this paper also demonstrated that NBTI is the most important aging mechanism for 28-nm FPGAs. Moreover, it is also worth noting that there were two other contributions of this paper for the community: the error correction method for the aging test and the prediction of FPGA aging based on machine learning models.

To reduce the duration of FPGA aging tests, common practice is to place the device in a high-temperature test box to accelerate aging. However, due to the measurement errors that are caused by high-temperature environments, the delay that is measured does not reflect the actual degree of aging degradation of the device. To this end, we proposed an error correction method for the aging tests. Our experiments showed that the error correction method proposed in this paper is effective. In addition, as far as the literature that was reviewed by the authors is concerned, there are few studies on predicting FPGA aging based on ML. To evaluate the aging trends of devices more efficiently, we explored the use of machine learning models to build an FPGA aging prediction model. Through experimental evaluation, the aging prediction model that was based on machine learning can better fit the real aging trends of devices.

## 5. Conclusions and Future Work

In this work, we studied the main aging mechanisms of 28-nm FPGAs. Different stress signals and LUT configurations were applied in aging tests. The results showed that NBTI is the main reason for FPGA aging degradation. To collect accurate aging data, we further analyzed the influence of temperature on measurement errors and proposed an error correction method. The results showed that the difference between the corrected measurement results and the simulation results was less than 1%, thereby proving that the correction method is efficient. Moreover, we employed four ML models that were trained using aging data to predict FPGA aging. Among them, the mean error rate of the XGBoost prediction was only 0.292%, which proves that it would be very feasible to use the ML model to predict the aging trends of FPGAs.

In future work, we will evaluate the effectiveness of the error correction and aging prediction methods that were proposed in this paper more comprehensively by testing different types of FPGAs. In addition, we will investigate more age-related features (e.g., failure rate) and incorporate them into the prediction models to further improve the accuracy of the model prediction. For the established aging prediction model, we will apply it to preventive maintenance in order to evaluate and predict the trends and extent of the circuit aging of FPGAs under different stress signals and LUT configurations. This will support the rational use of age-aware scheduling strategies to achieve aging mitigation.

## Figures and Tables

**Table 1:** Conditions of accelerated tests.

Factor	Relationship	Stress Condition	Acceleration
Core Voltage Supply	$t_{f}\propto V_{gs}^{-\gamma }$	1.1 V	≈10×
Temperature	$t_{f}\propto exp(\frac{E_{a}}{kT})$	373 K	≈2×
Voltage and Temperature			≈20×

**Table 2:** The results of λ.

Coefficient	$\lambda_{1}$	$\lambda_{2}$	$\lambda_{3}$	$\lambda_{4}$	$\lambda_{5}$	$\lambda_{6}$
**Value**	0.0087	0.0083	0.0069	0.0074	0.0063	0.0063
**Coefficient**	** $\lambda_{7}$ **	** $\lambda_{8}$ **	** $\lambda_{9}$ **	** $\lambda_{10}$ **	** $\lambda_{11}$ **	** $\lambda_{12}$ **
**Value**	0.0053	0.0038	0.0033	0.0027	0.0016	0.0001

**Table 3:** The results of λ over time.

Coefficient	100 h	200 h	300 h	400 h	500 h
$\lambda_{1}$	0.0074	0.0076	0.0075	0.0074	0.0075
$\lambda_{2}$	0.0038	0.0039	0.0038	0.0039	0.0038
$\lambda_{3}$	0.0000	0.0002	0.0000	0.0001	0.0000
**Coefficient**	**600 h**	**700 h**	**800 h**	**900 h**	**1000 h**
$\lambda_{1}$	0.0075	0.0074	0.0074	0.0074	0.0074
$\lambda_{2}$	0.0038	0.0040	0.0038	0.0039	0.0038
$\lambda_{3}$	0.0000	0.0000	0.0001	0.0000	0.0000
